# The TSIS‐1 Hybrid Solar Reference Spectrum

**DOI:** 10.1029/2020GL091709

**Published:** 2021-06-14

**Authors:** O. M. Coddington, E. C. Richard, D. Harber, P. Pilewskie, T. N. Woods, K. Chance, X. Liu, K. Sun

**Affiliations:** ^1^ Laboratory for Atmospheric and Space Physics University of Colorado Boulder Boulder CO USA; ^2^ Department for Atmospheric and Oceanic Science University of Colorado Boulder Boulder CO USA; ^3^ Harvard‐Smithsonian Center for Astrophysics Cambridge MA USA; ^4^ Department of Civil, Structural and Environmental Engineering University at Buffalo Buffalo NY USA; ^5^ Research and Education in ENergy, Environment and Water (RENEW) Institute University at Buffalo Buffalo NY USA

**Keywords:** High accuracy, high resolution, new reference spectrum, solar irradiance

## Abstract

We present a new solar irradiance reference spectrum representative of solar minimum conditions between solar cycles 24 and 25. The Total and Spectral Solar Irradiance Sensor‐1 (TSIS‐1) Hybrid Solar Reference Spectrum (HSRS) is developed by applying a modified spectral ratio method to normalize very high spectral resolution solar line data to the absolute irradiance scale of the TSIS‐1 Spectral Irradiance Monitor (SIM) and the CubeSat Compact SIM (CSIM). The high spectral resolution solar line data are the Air Force Geophysical Laboratory ultraviolet solar irradiance balloon observations, the ground‐based Quality Assurance of Spectral Ultraviolet Measurements In Europe Fourier transform spectrometer solar irradiance observations, the Kitt Peak National Observatory solar transmittance atlas, and the semi‐empirical Solar Pseudo‐Transmittance Spectrum atlas. The TSIS‐1 HSRS spans 202–2730 nm at 0.01 to ∼0.001 nm spectral resolution with uncertainties of 0.3% between 460 and 2365 nm and 1.3% at wavelengths outside that range.

## Key Points

- The TSIS‐1 Spectral Irradiance Monitor and Compact SIM instruments observe the Sun's irradiance spectrum at high accuracy
- The TSIS‐1 Hybrid Solar Reference Spectrum consists of high resolution solar line data normalized to the TSIS‐1 SIM irradiance spectrum
- The TSIS‐1 Hybrid Solar Reference Spectrum has at least 0.01 nm spectral resolution, spans 202–2730 nm, and is accurate to 0.3%–1.3%

## 1 Introduction

Reference solar irradiance spectra have broad utility in atmospheric science and climate applications. For example, the solar spectral irradiance (SSI) is used to convert measured satellite radiance to reflectance (e.g., Wielicki et al., 2013) and as the upper boundary condition in radiative transfer models used, for example, in remote sensing algorithms and renewable energy research (e.g., Apell & McNeill, 2019; Berk et al., 2014). Some instruments use solar absorption lines for wavelength calibration (e.g., Kang et al., 2020). Some instruments also use the Sun for radiometric stability monitoring, which requires a baseline solar spectrum to quantify instrumental changes against (e.g., Pan & Flynn, 2015). Instruments that monitor radiometric calibration stability relative to the moon (e.g., Werdell et al., 2019) indirectly rely on a solar reference spectrum to convert lunar radiance to reflectance using, for example, the RObotic Lunar Observatory (ROLO) model (Kieffer & Stone, 2005). Solar reference spectra also constrain solar irradiance variability models (e.g., Coddington et al., 2016), which climate models use to specify solar forcing of climate change (e.g., Kunze et al., 2020).

Various solar reference spectra exist for these applications. Some are from direct solar irradiance observations from one or more satellite instruments. These have relatively high reported accuracy and relatively low (0.1 nm or poorer) spectral resolution compared to ground‐based observations and are typically specific to certain solar activity levels (e.g., Thuillier et al., 2004). Other solar reference spectra are constructed by normalizing high spectral resolution solar lines to a higher accuracy, lower resolution, spectrum (e.g., Dobber et al., 2008). Still others are created by concatenating independent datasets from different spectral regions (e.g., Gueymard, 2003). Disagreements have been identified between the available solar reference spectra and independent measurements that exceed quoted accuracies particularly at near‐infrared wavelengths where 8% differences have been reported (e.g., Elsey et al., 2017).

Since March 2018, NASA's Total and Spectral Solar Irradiance Sensor‐1 (TSIS‐1) Spectral Irradiance Monitor (SIM) hosted on the International Space Station (ISS) has observed SSI with lower radiometric uncertainty (<0.3%) over the majority of the spectrum than that attained by previous instruments (Richard et al., 2020). Since 2019, independent SSI observations have also been made by the CubeSat Compact SIM (CSIM) instrument (Richard et al., 2019; Tomlin et al., 2020). CSIM observations span 200 –2800 nm, thereby extending further into the infrared than the TSIS‐1 SIM that spans 200 –2400 nm. A mutual validation of the TSIS‐1 SIM and CSIM irradiance scales was demonstrated by <1% disagreement in concurrent observations (Stephens et al., 2020). In this work, we produce a new reference spectrum, the *TSIS‐1 Hybrid Solar Reference Spectrum* (*TSIS‐1 HSRS*), by adjusting high spectral resolution solar line data to the SI‐traceable irradiance scale of the TSIS‐1 SIM and CSIM instruments. Such an approach is necessary because the technology does not exist to measure the Sun's spectrum over a broad spectral range from a single instrument with both high accuracy and high (0.01 nm or finer) spectral resolution.

**Figure 1:**
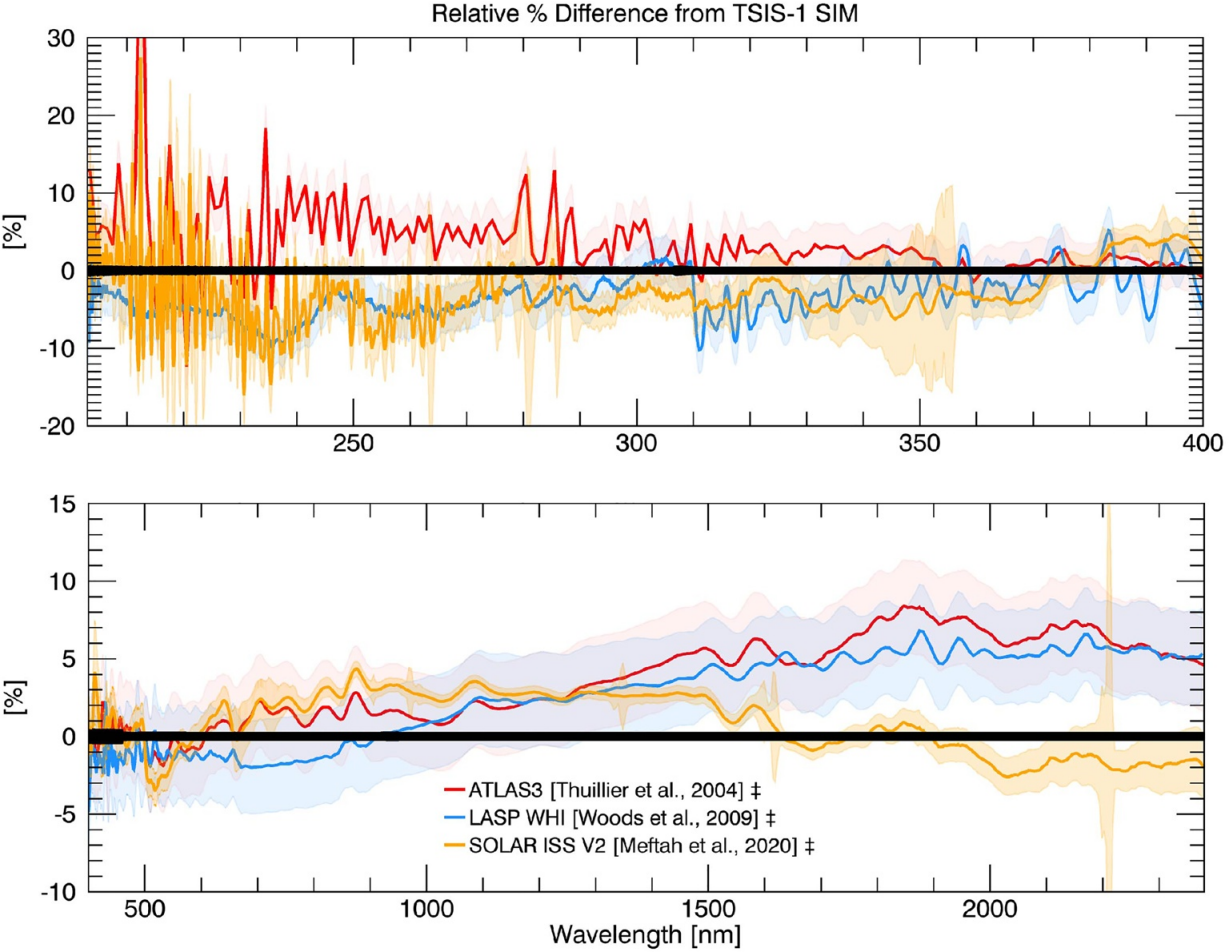
Percent relative difference between the ATLAS‐3, SOLAR‐ISS (v2) and LASP WHI solar reference spectra from TSIS‐1 SIM (see text). All datasets have been convolved to the TSIS‐1 SIM spectral resolution prior to computing the difference as (Reference–TSIS‐1 SIM)/TSIS‐1 SIM × 100.

Figure 1 shows spectral differences of three solar irradiance reference spectra to TSIS‐1 SIM of order 10% in portions of the spectrum and that cannot be explained by differences in solar activity. While ultraviolet solar cycle variability reaches 10% at 200 nm, it drops to 5% by 210 nm and reduces even further to ∼1% by 300 nm with the exception of the Mg II line near 280 nm. Visible and near‐infrared solar cycle variability is on the order of 0.1% or less (Ermolli et al., 2013). The ATLAS‐3 spectrum (Thuillier et al., 2004), perhaps the most widely used solar reference in Earth science applications, is a composite of observations from November 1994 by five different instruments including the SOLar SPECtrometer (SOLSPEC). Additionally, high resolution modeled solar absorption features from Kurucz (1995) were inserted into the lower resolution observations from the visible through the near‐infrared. Reported ATLAS‐3 uncertainties are 2%–3%. Another solar reference spectrum is the Laboratory for Atmospheric and Space Physics (LASP) Whole Heliospheric Interval (WHI) (Woods et al., 2009). The LASP WHI is a composite of observations from April 2008 with the majority of the spectrum measured by the SOLSTICE and SIM instruments on the SORCE satellite. Observations from SORCE SIM, the predecessor to the TSIS‐1 SIM, were adjusted by up to +8% for wavelengths above 1350 nm to agree with the ATLAS‐3 spectrum in a recalibration that has been discussed with reference to a systematic bias (Harder et al., 2010). Therefore, the LASP WHI and ATLAS‐3 reference spectra are not independent above 1350 nm. Reported LASP WHI uncertainties are 1%–3% for wavelengths above 300 nm. The SOLAR‐ISS version 2 reference spectrum (Meftah et al., 2020) is from a newer version of the SOLSPEC instrument (Thuillier et al., 2009) integrated on the ISS from 2008 to 2017. The SOLAR‐ISS reference irradiance baseline spectrum is from April 2008 for wavelengths spanning 165–656 nm and an average over a six year period at wavelengths above 656 nm. Revised engineering corrections, improved calibrations, and advanced thermal and degradation corrections are reported as the reason for the changes in baseline between the earlier ATLAS‐3 composite spectrum and the newer SOLAR‐ISS spectrum (Bolsée et al., 2017). However, particularly in the near‐infrared, a thorough understanding of the offset remains under study (Thuillier et al., 2013). Similar to ATLAS‐3, higher spectral resolution lines have been incorporated into SOLAR‐ISS. The mean reported SOLAR‐ISS uncertainty from 165 to 3000 nm is 1.26%, with uncertainties as low as 0.4%–0.6% between 800 to 1700 nm and reaching, or exceeding, 2% below 400 nm and above 2200 nm. Hilbig et al. (2018) further summarize these and other solar reference spectra.

The methodology to develop the HSRS is described in Section 2 and the datasets are described in Section 3. In Section 4, we present results of our uncertainty assessment and comparison to independent datasets. Concluding statements follow in Section 5.

## 2 Methodology

We develop the HSRS using a modified version of the spectral ratio method. In this method, a wavelength‐dependent scaling factor adjusts high spectral resolution datasets (β) to match a lower resolution but higher accuracy spectrum (α). The scaling factor, *Q*, is the ratio of the α and β datasets after first convolving both to the same spectral resolution and interpolating to a common sampling grid. The α and β datasets are described in Section 3.

Typically, *Q* is derived after a single‐step convolution (Equation 1) of the β data set with the instrument line shape of the α data set ($\mathrm{IL}S_{\alpha }$) that degrades the resolution of the β data set ($\beta^{*}$) to match that of the α data set (e.g., Dobber et al., 2008; Kang et al., 2017). Instead, we derive *Q* from a two‐step convolution: The first step is as described by Equation 1 and the second step degrades both $\beta^{*}$ and α datasets to a common spectral resolution (denoted $\beta^{**}$ and $\alpha^{**}$, respectively) that is coarser than that of the original α data set. We accomplish this with a Gaussian filter (ℕ) of specified standard deviation (σ) (Equation 2). The two‐step convolution reduces the impacts of any uncertainty in $\mathrm{IL}S_{\alpha }$ on *Q* (Equation 3), where the subscript ‡ denotes an interpolation of the $\beta^{**}$ data set to the $\alpha^{**}$ sampling grid. Finally, the adjusted β data set (denoted by Υ) is computed from the product of the native β data set and *Q*, where the subscript † denotes an interpolation of *Q* to the native β sampling grid (Equation 4).

(1) $$\beta^{*}=\;\beta ⊗\mathrm{IL}S_{\alpha }$$

(2) $$\beta^{**}=\;\beta^{*}⊗\mathbb{N}\left(\sigma \right)\;and\;\alpha^{**}=\alpha ⊗\mathbb{N}\left(\sigma \right)$$

(3) $$Q=\alpha^{**}/\beta_{‡}^{**}$$

(4) $$\Upsilon =\beta \times Q_{†}$$

Υ represents the β datasets at the α spectrum irradiance scale. Υ datasets differ from the β datasets in their broad baseline features, but share the same native spectral and sampling resolutions. The TSIS‐1 HSRS is the concatenation of these Υ datasets. In transition regions, where one Υ data set overlaps in wavelength with another, we adopt an average of the irradiance values for the HSRS.

## 3 Data

### 3.1 High Accuracy (*α*) Spectrum

Our high accuracy α spectrum is space‐based SSI observations from the TSIS‐1 Spectral Irradiance Monitor (SIM) and Compact SIM (CSIM). TSIS‐1 SIM has measured daily SSI between 200‐2400 nm since March 2018. The CSIM data set, spanning 210–2800 nm, began in late‐March 2019. The SIM instruments have variable spectral resolution of approximately 0.25–40 nm (Richard et al., 2019; 2020). TSIS‐1 SIM and CSIM data are available from: https://lasp.colorado.edu/home/tsis/data/ssi-data/ and https://lasp.colorado.edu/home/csim/data-and-ham-radio/.

TSIS‐1 SIM meets climate‐record quality requirements (NOAA, 2010) and has order‐of‐magnitude reductions in radiometric uncertainty relative to the heritage SORCE SIM instrument (Harder et al., 2005) through an extensive component level calibration program that characterized the instrument as an absolute sensor and verified the instrument in irradiance across the spectrum against an SI‐traceable cryogenic radiometer using stable tunable laser sources (Richard et al. 2020). The instrument level validation and final end‐to‐end absolute calibration placed relative pre‐launch accuracy uncertainties at 0.24% (>460 nm) to 0.41% (<460 nm). On‐orbit calibration stability is maintained by instrument degradation corrections that utilize observations made by redundant and independent instrument channels that are exposed to the Sun at varying duty cycles (Mauceri et al., 2020). Precision is 0.01%–0.05% (Richard et al., 2020).

CSIM is a 6U CubeSat technology demonstration mission for the NASA Earth Science Technology Office. CSIM radiometric accuracy is tied to the same SI‐traceable cryogenic radiometer with the same laser sources used in the TSIS‐1 SIM calibrations, but by calibration transfer as opposed to absolute calibration verification. The CSIM measurement uncertainty is <1% from 300‐2000 nm and 1.26% above 2000 nm (Richard et al., 2019).

Specifically, the α spectrum, from 200 to 2365 nm, is an average of daily TSIS‐1 SIM irradiance observations from 1 to 7 December 2019, which coincides with the solar activity minimum between solar cycles 24 and 25 (https://www.swpc.noaa.gov/news/solar-prediction-scientists-announce-solar-cycle-25). We extend this spectrum from 2365 to 2730 nm with averaged CSIM observations from April to September 2019. A wavelength‐independent offset factor of 0.9921 (i.e., 0.8%; within the measurement uncertainty) ensures the CSIM irradiance portion of the α spectrum (i.e., ≥2365 nm) matches TSIS‐1 SIM irradiance at 2365 nm.

### 3.2 High Spectral Resolution (*β*) Datasets

The β datasets are the Air Force Geophysical Laboratory (AFGL) solar irradiance observations, the Kitt Peak National Observatory (KPNO) solar transmittance atlas, the Quality Assurance of Spectral Ultraviolet Measurements In Europe (QASUME) Fourier transform spectrometer (QASUMEFTS) solar irradiance observations, and the Solar Pseudo‐Transmittance Spectrum (SPTS) atlas.

Grating spectrometer observations of the Sun's ultraviolet irradiance from high‐altitude balloons dating to the 1970s and 1980s by the Air Force Geophysical Laboratory (AFGL) (Hall & Anderson, 1991) are the only SSI data set available to date between 200  and 310 nm with a spectral resolution of 0.01 nm or better. Corrections for atmospheric ozone absorption attenuation were applied to the data. The spectral and sampling resolution of the AFGL irradiance data set are 0.01 nm and the radiometric uncertainty is typically 5%–10%, but can reach 25% near 200 nm.

Additional high resolution data are solar transmittances between 300 and 1000 nm (Kurucz, 2005) derived from Kitt Peak National Observatory (KPNO) ground‐based Fourier transform spectroradiometer (FTS) observations between 296 and 1300 nm at ∼0.001 nm resolution (Kurucz et al., 1984). Converting from FTS observation to transmittance was achieved through a multi‐step process (Kurucz, 2005). First, continuum atmospheric absorption features based were removed based on a model followed by the estimation and removal of the solar continuum with subjective fits of the FTS observations to a simulated solar spectrum. Sharp telluric spectral features, attributed to molecules in Earth's atmosphere, were identified with the HITRAN database (Rothman et al., 2005) and removed. The KPNO residual irradiance wavelength scale accuracy, reassessed for this study, is found to be better than 3.2 × 10^−4^ nm above 305 nm and better than 3.0 × 10^−3^ nm at shorter wavelengths, unchanged from that reported in Chance and Kurucz (2010).

An additional source is the high‐resolution extraterrestrial solar irradiance spectrum measured by an FTS between 305  and 380 nm from a high‐altitude, ground location during the Quality Assurance of Ultraviolet Measurements In Europe (QASUMEFTS) campaign. The measured spectrum was extended down to 300 nm and up to 500 nm with the KPNO atlas (Gröbner et al., 2017). The extraterrestrial solar spectrum was derived from QASUME observations by the Langley plot technique. The FTS observations were adjusted to the absolute irradiance scale of a lower‐resolution, reference spectroradiometer with accuracy traceable to the primary spectral irradiance standard of the Physikalisch Technische Bundesanstalt (PTB) laboratory in Germany. QASUMEFTS radiometric uncertainty (*k* = 2) reaches 4% at wavelengths lower than 310 nm and 2% between 310 and 500 nm. The spectral resolution of QASUMEFTS is better than 0.025 nm and uncertainty in the wavelength‐scale is 0.01 nm or better.

Version 2016 of the “disk‐integrated” Solar Pseudo‐Transmittance Spectrum (SPTS) (Toon, 2014) contains the transmittance from 40,000 solar absorption lines spanning 600‐26,316 cm^−1^ (380–16,600 nm), sampled every 0.01 cm^−1^. It is an empirically generated data set, where telluric line contributions to the observed spectra from multiple FTS instruments are identified with the HITRAN database and iteratively removed. Measured KPNO spectra are the predominant observation source in the SPTS database, supplemented with observations from high‐altitude balloons and satellites (Toon, 2013).

We adopt a vacuum wavelength scale for the HSRS. The AFGL and QASUMEFTS datasets were converted from air‐to‐vacuum scale using Edlén (1966).

## 4 Results

In this section, we present the TSIS‐1 Hybrid Solar Reference Spectrum (HSRS) and make comparisons to independent datasets.

### 4.1 *Q* Factor

When the spectral ratio method is used to adjust an *irradiance* data set, *Q* is unitless and represents a magnitude adjustment to the radiometric calibration of the original data set. However, when the method is applied to adjust a *solar transmittance* data set, *Q* has units of SSI (W/m^2^/nm) and approximates the solar continuum when devoid of absorption and emission features. In either case, *Q* adjusts broad features while leaving fine spectral features undisturbed.

**Figure 2:**
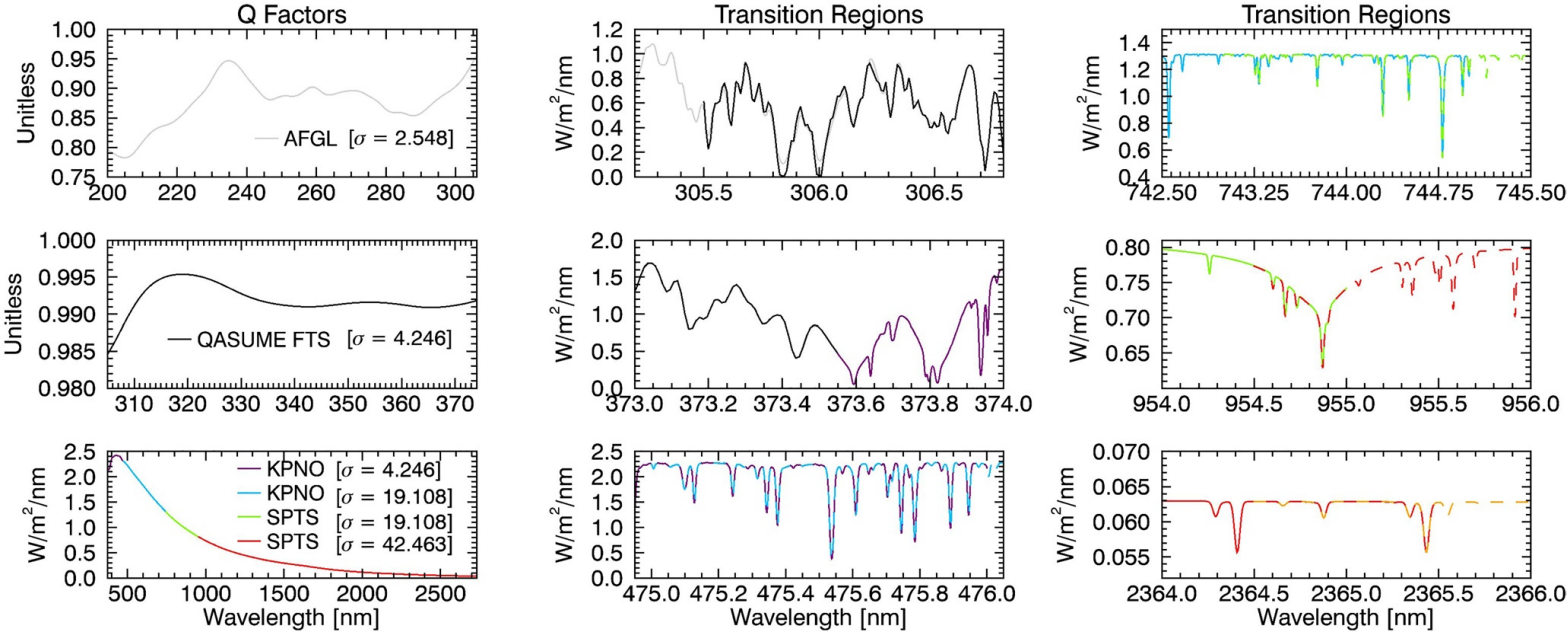
(left column) The *Q* factors used to adjust the AFGL, QASUMEFTS, KPNO, and SPTS datasets to the α spectrum radiometric scale over the spectral ranges shown using the Gaussian convolution filters of the standard deviation reported in the legend. (middle and right columns) Detailed comparisons of all transition regions in the datasets comprising the TSIS‐1 HSRS (see text) identified by the color of the legend.

Figure 2 shows the *Q* factors used to produce the HSRS at the α spectrum irradiance scale. The adjustments are smaller than 25% for AFGL and 2.5% for QASUMEFTS datasets, which falls within their respective reported radiometric uncertainties. The adjustments for the KPNO and SPTS solar transmittance datasets have the expected spectral shape of the Sun's continuum.

**Figure 3:**
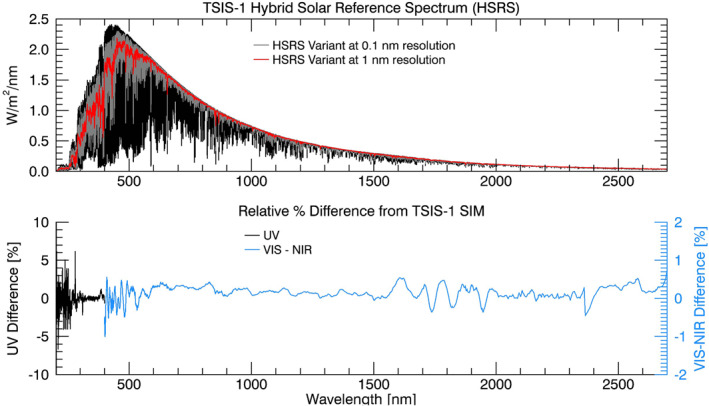
(top) The TSIS‐1 Hybrid Solar Reference Spectrum (black) and two variants at lower resolution. (bottom) The relative percent difference of the HSRS from the α spectrum, computed identically as in Figure 1, with separate percent difference *y*‐axis scales for the ultraviolet (UV; *λ* < 400 nm) and visible‐to‐near‐infrared (VIS‐NIR; *λ* > 400 nm) portions of the spectrum. Near‐identical results are obtained when computing the relative difference for the variants.

Applying *Q* to the β datasets forms the TSIS‐1 HSRS at 0.01 to ∼0.001 nm spectral resolution and spanning 202–2730 nm (Figure 3, top). We also produce four variants of the HSRS that standardize the reference spectrum to fixed, lower spectral resolutions using Gaussian convolution filters. The integrated SSI of the HSRS and the HSRS variants is within 0.2% of the integrated α spectrum between 202 and 2730 nm (1,324.94 W m^−2^). We produce an additional variant of the HSRS data set (not shown) over the spectral range 202–500 nm with variable Gaussian convolution kernels that approximate the spectral resolution, but not the true spectral shape, of the SORCE Solar‐Stellar Irradiance Comparison Experiment (SOLSTICE) (McClintock et al., 2005) and the Aura Ozone Monitoring Instrument (OMI) (Levelt et al., 2006). This final variant has utility for developing new, higher resolution, solar irradiance variability models (Lean et al., 2020). The HSRS and its variants are reported on fixed wavelength grids of at least four points per resolution element (Table 1).

**Table 1:** Summary of the HSRS Reference Spectra

File name	High resolution datasets and wavelength coverage (nm)	Spectral resolution	Sampling resolution	Uncertainty (%)
TSIS‐1 HSRS	AFGL: 202–306.5 QASUMEFTS: 305.5–373.6 KPNO: 373.5–745 SPTS: 743–2730	Varies; equal to that of the original high resolution, β, datasets	0.001 nm	<400 nm = 1.3 400–460 nm = 0.5 460–2365 nm = 0.3 >2365 nm = 1.3
TSIS‐1 HSRS “p005 nm”	Same as TSIS‐1 HSRS	0.025 nm (below 374 nm) 0.005 nm (above 374 nm)	0.001 nm	Same as above
TSIS‐1 HSRS “p025 nm”	Same as TSIS‐1 HSRS	0.025 nm	0.005 nm	Same as above
TSIS‐1 HSRS “p1nm”	Same as TSIS‐1 HSRS	0.1 nm	0.025 nm	Same as above
TSIS‐1 HSRS “1 nm”	Same as TSIS‐1 HSRS	1 nm	0.2 nm	Same as above
TSIS‐1 HSRS “SOL‐OMI”	AFGL: 202–309.8 QASUMEFTS: 306.4–373.5 KPNO: 373.3–500	0.048 nm (below 310 nm) 0.42 nm (310–360 nm) 0.62–0.64 nm (360–500 nm)	0.025 nm	<400 nm = 1.3 400–460 nm = 0.5 460–500 nm = 0.3

Column 2 is the β datasets spectral range, columns 3 and 4 are the spectral and sampling resolutions, and column 5 is the total uncertainty. The spectral resolution of the HSRS variants is defined by the full‐width half‐maximum value of the Gaussian convolution kernel.

### 4.2 Uncertainties

The total TSIS‐1 HSRS uncertainty (Table 1) is the root‐sum‐square of the following error sources: The uncertainties of the TSIS‐1 SIM and CSIM measurements that comprise the α spectrum, including those incurred from instrument degradation corrections, and the methodology accuracy. The methodology uncertainty is the 1*‐* σ standard deviation of the relative percent difference of the HSRS and the α spectrum computed separately for the UV (<400 nm) and VIS‐NIR (400–2365 nm) and long NIR (>2365 nm) portions of the spectrum (Figure 3; bottom) and equal to 1.2%, 0.16%, and 0.36%, respectively. The HSRS uncertainty is equivalent to 0.3% over most of the spectrum, increasing to 1.3% below 400 nm and above 2365 nm. It reflects the uncertainty of the HSRS *for the same spectral resolution as the TSIS‐1 and the CSIM instruments*. At very high spectral resolution, the relative differences in individual lines from different solar line databases can reach several tens of percent (not shown).

### 4.3 Comparison to Other Datasets

Figures 1 and 3 establish the difference between the HSRS and the ATLAS‐3 and LASP WHI solar reference spectra that is several percent between 500  and 1300 nm, increasing to 8%–10% for wavelengths outside of that range. The SOLAR‐ISS differs at individual wavelengths by −3.3% (∼−0.06 W m^−2^) near the peak of the solar spectrum at 520 nm and by +2 to +4% (−0.01 to −0.03 W m^−2^) between 800  and 1400 nm. Above 1500 nm, the agreement is generally within 2%. In the ultraviolet, differences between the HSRS and the other reference spectra can approach 10%.

**Figure 4:**
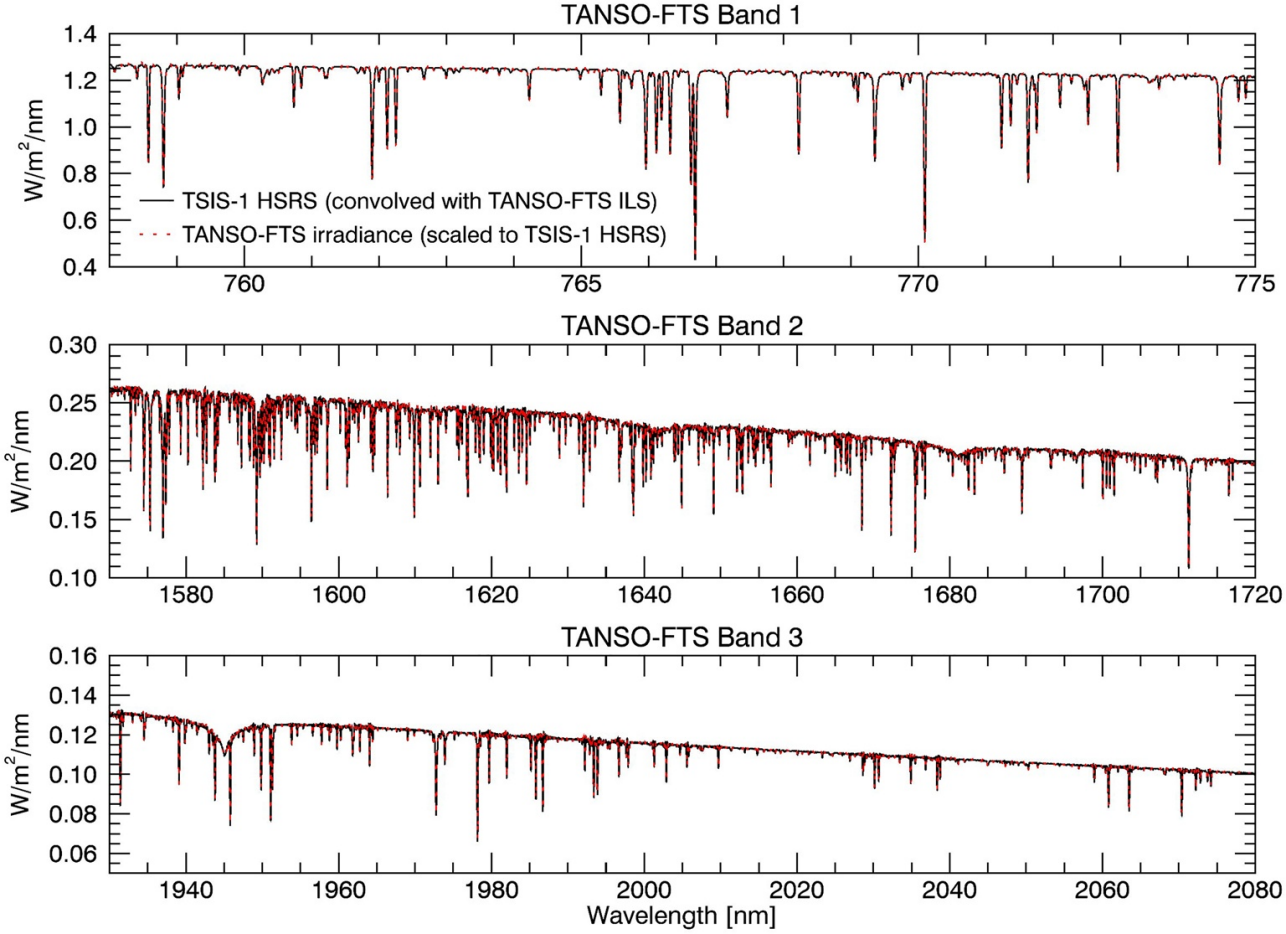
A comparison of the TSIS‐1 HSRS to a GOSAT TANSO‐FTS solar irradiance spectrum derived from solar radiances measured in three bands during calibration scans (see text).

In Figure 4, we compare the HSRS to high‐resolution TANSO Fourier Transform Spectrometer (TANSO‐FTS) observations obtained during solar calibration scans of the Greenhouse Gases Observing Satellite (GOSAT) mission (Kuze et al., 2009). For the comparison, the HSRS resolution has been reduced to match that of the TANSO‐FTS instrument. We also apply adjustments to the TANSO‐FTS data. First, we correct the wavelength scale for the Doppler shift that occurs with changing spacecraft velocity. Second, we convert the s‐ and p‐polarized solar radiance to solar irradiance under the assumption of a perfect solar diffuser plate. Third, we average the Doppler‐corrected, s‐ and p‐polarized irradiance to get the unpolarized solar irradiance spectrum. Finally, we adjust the irradiance scale to match that of the HSRS using the spectral ratio method described in Section 2. The resulting 1*‐* σ standard deviation of the HSRS and TANSO‐FTS relative percent difference is smaller than 0.4% in all bands (not shown), demonstrating robust HSRS solar line positions and depths in these wavelength ranges.

## 5 Conclusions

The TSIS‐1 Hybrid Solar Reference Spectrum (HSRS) is a new solar minimum irradiance reference spectrum developed by normalizing high spectral resolution solar line data to the absolute irradiance scale of the TSIS‐1 SIM and CSIM. TSIS‐1 SIM and CSIM observe SSI at higher accuracy than attained by predecessor instruments and, notably, show the near‐infrared solar spectrum is 8%–10% lower in magnitude than the ATLAS‐3 and LASP WHI reference spectra. The SOLAR‐ISS (v2) reference spectrum agrees with the TSIS‐1 SIM over most wavelengths above 1600 nm but disagreements persist from 500‐1600 nm. Differences can reach 10% below 300 nm. Therefore, the HSRS provides an important new constraint for science analyses in a broad array of fields.

The HSRS spans 202–2730 nm, encompassing an integrated energy that exceeds 97% of the total solar irradiance. The HSRS accuracy is 0.3%–1.3% and the spectral resolution is 0.01 –∼0.001 nm. Variants of the HSRS are also provided for lower, fixed spectral resolutions.

## Data Availability

The TSIS‐1 HSRS is available from https://lasp.colorado.edu/lisird/data/tsis1_hsrs.
